# Depressive Symptoms and Associated Factors Among Middle-Aged and Older Patients with Chronic Kidney Disease: Gender Differences Based on a Health Ecological Model

**DOI:** 10.3390/healthcare13161951

**Published:** 2025-08-09

**Authors:** Yu Zhang, Yingqi Huang, Wenhui Zhang, Ya Shi, Youtao Mou, Yuanyuan Lan, Manoj Sharma, Lei Zhang, Yong Zhao

**Affiliations:** 1 School of Public Health, Chongqing Medical University, Chongqing 400016, China; 3355622191@stu.cqmu.edu.cn (Y.Z.); 2022222041@stu.cqmu.edu.cn (W.Z.); 2023121694@stu.cqmu.edu.cn (Y.S.); 2024121761@stu.cqmu.edu.cn (Y.M.); 2024121736@stu.cqmu.edu.cn (Y.L.); 2 Research Center for Medicine and Social Development, Chongqing Medical University, Chongqing 400016, China; 3 Research Center for Public Health Security, Chongqing Medical University, Chongqing 400016, China; 4 Nutrition Innovation Platform-Sichuan and Chongqing, School of Public Health, Chongqing Medical University, Chongqing 400016, China; 5 College of International Education, Sichuan International Studies University, Chongqing 400031, China; 10202402200033@stu.sisu.edu.cn; 6 Department of Social and Behavioral Health, School of Public Health, University of Nevada, Las Vegas, NV 89154, USA; manoj.sharma@unlv.edu; 7 Department of Internal Medicine, Kirk Kerkorian School of Medicine, University of Nevada, Las Vegas, NV 89154, USA; 8 China-Australia Joint Research Center for Infectious Diseases, School of Public Health, Xi’an Jiaotong University Health Science Center, Xi’an 710061, China; lei.zhang1@monash.edu; 9 Artificial Intelligence and Modelling in Epidemiology Program, Melbourne Sexual Health Centre, Alfred Health, Melbourne 3053, Australia; 10 School of Translational Medicine, Faculty of Medicine, Nursing and Health Sciences, Monash University, Melbourne 3800, Australia; 11 Chongqing Key Laboratory of Child Nutrition and Health, Children’s Hospital of Chongqing Medical University, Chongqing 400014, China

**Keywords:** depressive symptoms, chronic kidney disease, older adults, CHARLS, health ecology-based model

## Abstract

**Objectives**: Depressive symptoms are highly prevalent among individuals with chronic kidney disease (CKD). This study explores their associated factors and gender differences among middle-aged and older CKD patients in China. **Methods**: Based on the health ecology model (HEM), this study utilized the 2018 cross-sectional data from the China Health and Retirement Longitudinal Study (CHARLS) to examine gender differences in CKD patients across demographic groups. A multivariate logistic regression identified factors associated with depressive symptoms and gender differences among middle-aged and older patients with CKD in China. Additionally, a random forest model was constructed to rank the importance of key predictors based on the Gini index. **Results**: Among 1422 CKD patients, 50.35% reported depressive symptoms (42.97% of males and 59.56% of females). Factors significantly associated with higher depressive symptoms included female gender, rural residence, poor self-reported health, sleep duration < 7 h, and limitations in Activities of Daily Living (ADLs) and Instrumental Activities of Daily Living (IADLs). The association of smoking and ADLs on depressive symptoms in CKD patients varied considerably between genders. Self-reported health and life satisfaction were the two variables most strongly associated with depressive symptoms among CKD patients. **Conclusions**: The study shows that female CKD patients have a higher prevalence of depressive symptoms than males. Several factors are significantly associated with depressive symptoms in patients with CKD. These findings provide valuable insights that potentially inform the development of targeted prevention and management strategies for depressive symptoms in middle-aged and older CKD patients in China.

## 1. Introduction

Depression is a psychological disorder characterized by persistent sadness, sleep and appetite disturbances, lack of concentration, and reduced self-esteem. In severe cases, it may result in self-harm or suicidal behaviors, with depressive symptoms being one of its primary manifestations [1,2]. Studies have found that the prevalence of depressive symptoms ranges from 10.3% to 13.6% in high-income Western countries [3,4], while in Asian nations, such as India and Thailand, the prevalence of depressive symptoms falls between 19.4% and 46% [5,6]. In China, 24.1% of individuals aged 45 and above exhibit depressive symptoms [1]. According to the Global Burden of Disease (GBD) report, depression ranks as the second-leading cause of disease burden in China [7], as well as the second-largest contributor to rising disability-adjusted life years (DALYs), profoundly impairing the well-being of middle-aged and older individuals [8].

Chronic kidney disease (CKD) is characterized by irreversible kidney damage that persists for more than three months [9]. Between 1990 and 2017, the global prevalence of CKD increased by 38.4%, affecting approximately 700 million individuals [10]. During this period, mortality due to CKD rose to 41.5% [11]. In China, the prevalence of CKD is estimated at 8.2%, with over 60% of cases occurring among middle-aged and older individuals [10,12,13]. CKD is associated with a range of adverse outcomes, including psychological disorders, renal failure, and premature mortality [14]. Systematic reviews indicate that depressive symptoms are prevalent mental health symptoms among CKD patients, affecting approximately one-quarter of this population [15]. Among treated CKD patients, the highest reported prevalence of depressive symptoms is 39.3% [15]. A meta-analysis further demonstrated that depressive symptoms are significantly independently associated with disease progression in adults with end-stage renal disease undergoing long-term dialysis treatment [16]. Moreover, depressive symptoms have been associated with an increased risk of mortality among patients with CKD [16], underscoring the substantial health burden imposed by depressive symptoms in this population. In a study by Liu, L. et al., it was found that the prevalence of depressive symptoms among female CKD patients in the United States was nearly twice that of male patients, with a significant difference observed [17]. Similarly, gender differences in the prevalence of depressive symptoms have also been identified in middle-aged and older populations in China [18]. Previous research suggests that factors such as social support, life satisfaction, and physical health status are associated with the presence of depressive symptoms in older adults [19,20,21]. Therefore, it is necessary to describe and explore the factors associated with depressive symptoms and gender differences among middle-aged and older CKD patients in China. In addition, previous studies have predominantly employed traditional logistic regression models for data analysis. Logistic regression is primarily used to examine the association between categorical outcomes and multiple influencing factors and is valued for its interpretability [22]. However, with the increasing application of machine learning techniques in medical research, the random forest algorithm has emerged as an important tool in clinical studies due to its superior classification performance and intuitive learning mechanism. Existing studies have demonstrated that, compared to other machine learning algorithms, random forest offers notable advantages in handling missing data, enhancing model interpretability, and facilitating practical implementation [23].

The health ecology model (HEM) posits that the social environment exerts multi-level and complex influences on individuals. It emphasizes that both individual and population health are shaped by the interrelation and interplay of demographics, health behavior, social network, living and working conditions, and social policy [24,25]. These factors are interdependent and collectively contribute to a balanced health ecosystem. This study applied the HEM framework to investigate depressive symptoms and their associated factors, with a focus on gender differences, among middle-aged and older CKD patients in China. A random forest model was utilized to identify key factors, aiming to provide precise information for the intervention and management of depressive symptoms in CKD patients of different genders.

## 2. Materials and Methods

### 2.1. Participants and Process

The data utilized in this study were obtained from the China Health and Retirement Longitudinal Study (CHARLS) [26]. CHARLS is a nationally representative survey designed to collect reliable data on individuals and households aged 45 and above in China. The initial baseline survey was conducted in 2011, followed by four successive national follow-up surveys. The survey employed a multi-stage probability sampling method with proportional allocation to ensure a randomized and representative sample. Data were collected through face-to-face interviews conducted by trained staff using computer-assisted techniques, thereby enhancing the reliability and validity of the data. Informed consent was obtained from all participants before data collection. The CHARLS survey project has been approved by the Ethics Committee of Peking University (IRB00001052–11015). The CHARLS dataset can be accessed and downloaded for free after registration at http://charls.pku.edu.cn/ (accessed on 8 August 2022).

The sample size for this cross-sectional study was determined based on the formula: [n = (Z^2^_α/2_p q)/δ^2^] [27,28], where (1) n represents the required sample size for the study; (2) p is the prevalence of depressive symptoms among middle-aged and older CKD patients in China; (3) q = (1 − p); (4) Z_α/2_ is set to 1.96, corresponding to a significance level α of 0.05 for a two-sided test; (5) δ denotes the allowable error, calculated as 0.1p.

According to previous studies, the prevalence of depressive symptoms among middle-aged and older patients with CKD in China ranges from 23% to 37.8% [29,30]. To ensure sufficient statistical power and a conservative estimate of sample size, the prevalence was set at the lower bound of 23%. Based on this assumption and the sample size calculation formula, the present study requires a sample of 1286 participants.

**Figure 1:**
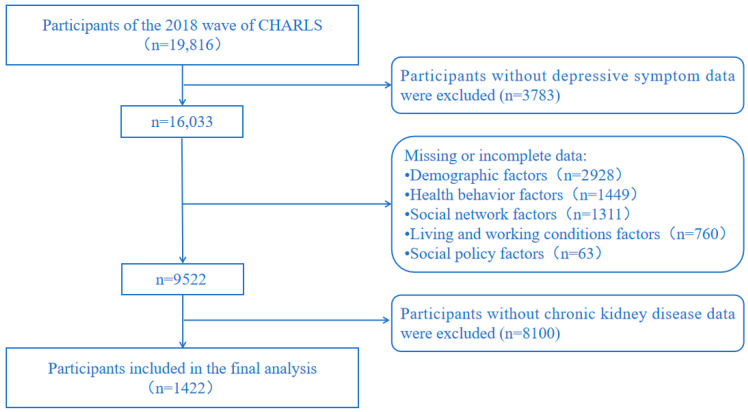
Flowchart of participant selection in the study.

This study utilized data from the 2018 wave of CHARLS, which covers 30 provinces and 150 districts, with a total of 19,816 respondents. The inclusion criteria for this study were as follows: (1) respondents aged 45 years and above; (2) no missing or invalid data on depressive symptoms; (3) no missing or invalid data on key covariates; (4) respondents diagnosed with CKD by a physician. Ultimately, 1422 individuals participated in the study. The process of sample refinement is depicted in Figure 1.

### 2.2. Assessment of CKD

CKD was determined based on self-reported physician diagnoses provided by respondents [31]. Participants were asked, “Have you been diagnosed with Kidney disease (except for tumor or cancer) by a doctor?” Respondents who answered “yes” were classified as having CKD. CKD patients in this study included those newly diagnosed in 2018, as well as those who were followed up from previous years.

### 2.3. Assessment of Depressive Symptoms

The Centre for Epidemiological Studies Depression Scale (CES-D-10) was utilized to evaluate the respondents’ depressive symptoms. Previous studies have demonstrated that the CES-D-10 exhibits good predictive accuracy and shows strong reliability and validity in studies involving middle-aged and older adults in China [32,33,34]. The CES-D-10 utilizes a 4-point Likert scale, with response options ranging from “3 = most of the time,” “2 = sometimes or about half of the time,” “1 = not too much,” to “0 = rarely or never.” The scale consists of 10 items, with items 5 (“I felt optimistic about the future”) and 8 (“I experienced happiness”) being reverse-scored. The total score ranges from 0 to 30, with higher scores indicating more severe depressive symptoms. An individual with depressive symptoms is considered to have a score of ≥10, which is the cutoff score based on earlier studies [35,36]. Cronbach’s alpha coefficient for the CES-D-10 is 0.819, demonstrating good internal consistency.

### 2.4. Covariates

The HEM is an extension of the ecological model, which emphasizes that an individual’s health is shaped by dynamic interactions with their surrounding environment. This model advocates analyzing the factors associated with health or disease from five perspectives: demographics, health behavior, social network, living and working conditions, and social policy [37]. By integrating health information from these diverse dimensions, the model ultimately aims to promote health. To ensure the reliability and comprehensiveness of the study findings, this research selected 24 covariates based on the HEM across five dimensions [38]: (1) Demographic factors: gender, age, ethnicity, self-reported health, hypertension, diabetes, dyslipidemia, stroke, activities of daily living (ADLs), and instrumental activities of daily living (IADLs); (2) Health behavior factors: smoking, drinking, sleep duration, nap time, and social activities; (3) Social network factors: marital satisfaction, marital status, children satisfaction, and life satisfaction; (4) Living and working conditions factors: education level, place of residence, and type of residence; (5) Social policy factors: pension and insurance. Detailed information on the questionnaire items and coding rules is provided in Table S1 of the Supplementary Materials.

CHARLS uses the ADL scale and the IADL scale to assess the ability to perform ADLs and IADLs, respectively [37]. ADLs include tasks such as putting on clothes, taking a bath or shower, having meals, moving in and out of bed, using the restroom, and managing bladder and bowel movements. IADLs include tasks such as cleaning the house, cooking meals, making phone calls, running errands, taking medications, and handling finances. Each item is rated on a scale of 0 to 1, where “0” indicates no difficulty, and “1” denotes some difficulty or complete inability to perform the activity. The total scores for ADLs and IADLs are calculated separately, ranging from 0 to 6 for each scale. A score of 0 indicates no limitations in ADL/IADL activities [39,40].

### 2.5. Statistical Methods

The study data were processed and analyzed using SPSS 26.0. To enhance the representativeness and robustness of the estimates, sampling weights were applied throughout the analysis. All variables in this study were categorical and presented as frequencies and percentages (n (%)). Multivariate logistic regression analysis was employed to examine the factors associated with depressive symptoms among middle-aged and older CKD patients in China. Additionally, we explored the prevalence of depressive symptoms by gender and analyzed their association with various demographic characteristics. Multivariate logistic regression analyses were conducted for depressive symptoms in CKD patients stratified by gender. In the multivariate logistic regression analyses, depressive symptoms were considered the outcome variable, while the five dimensions of the HEM were included as covariates for interaction analysis to examine their differential impact on depressive symptoms among male and female CKD patients. The Hosmer–Lemeshow test was used to evaluate the goodness-of-fit of the multivariate logistic regression model. We assessed potential multicollinearity among predictor variables by calculating Variance Inflation Factors (VIFs). VIF values less than 10 were interpreted as indicating an acceptable level of multicollinearity [41,42].

Finally, a random forest model was constructed using R 4.3.0 to rank variable importance in the overall sample and by gender. Grid search combined with 10-fold cross-validation was employed to optimize hyperparameters. This algorithm captures complex nonlinear relationships and interactions without requiring a predefined functional form, is robust to multicollinearity, and handles high-dimensional data well. Model performance was evaluated using the area under the receiver operating characteristic curve, accuracy, sensitivity, specificity, positive predictive value (PPV), and negative predictive value (NPV) [23].

A *p*-value below 0.05 was considered statistically significant.

## 3. Results

### 3.1. The Characteristics of Study Participants

Among the 1422 respondents, 76.44% were aged 60 years and above, 91.70% were of Han ethnicity, and 70.39% resided in rural areas. A total of 89.24% were married and cohabiting, and 15.89% were illiterate. The prevalence of depressive symptoms in Chinese middle-aged and older patients with CKD was 50.35%, with a depressive symptoms prevalence of 42.97% among male patients and 59.56% among female patients, as shown in Table 1.

### 3.2. Associated Factors of Depressive Symptoms in Chinese Middle-Aged and Older CKD Patients

Table 2 presents the results of a multivariate logistic regression analysis of depressive symptoms in 1422 Chinese middle-aged and older patients with CKD. The analysis reveals several significant risk factors for depressive symptoms: female patients (OR: 1.40, 95%CI: 1.05–1.87), rural areas (OR: 1.83, 95%CI: 1.32–2.54), poor self-reported health (OR: 3.04, 95%CI: 1.92–4.81), sleep duration < 7 h (OR: 1.37, 95%CI: 1.01–1.88), ADL limitations (OR: 1.48, 95%CI: 1.08–2.05), and IADL limitations (OR: 1.98, 95%CI: 1.46–2.68). Conversely, protective factors for depressive symptoms included being married and cohabiting (OR: 0.46, 95%CI: 0.30–0.70), nap time of ≥90 min (OR: 0.57, 95%CI: 0.37–0.87), marital satisfaction (OR: 0.33, 95%CI: 0.20–0.56), satisfaction with children (OR: 0.39, 95%CI: 0.19–0.77), and life satisfaction (OR: 0.22, 95%CI: 0.14–0.35). All reported associations were statistically significant (*p* < 0.05). As presented in Table 2, all VIF values were below the threshold of 10, indicating the absence of significant multicollinearity in the logistic regression model.

### 3.3. Description of the Basic Characteristics of Participants by Gender

Among Chinese middle-aged and older CKD patients, the prevalence of depressive symptoms varies by gender and is associated with sociodemographic characteristics, lifestyle and behavioral factors, and health status. As shown in Table S2 in the Supplementary Materials, among male CKD patients, significant differences in depressive symptoms are associated with self-reported health, residence, alcohol consumption, stroke, ADLs, IADLs, pension, activity, marital status, marital satisfaction, children’s satisfaction, and life satisfaction (*p* < 0.05). In female CKD patients, significant correlates include self-reported health, dyslipidemia, stroke, nap time, sleep duration, marital status, residence, education level, ADLs, IADLs, pension, marital satisfaction, children’s satisfaction, and life satisfaction (*p* < 0.05).

### 3.4. Gender Differences in Depressive Symptoms and Associated Factors Among CKD Patients

Table 3 reveals significant gender-specific risk and protective factors for depressive symptoms among Chinese middle-aged and older CKD patients. For male patients, rural areas (OR: 1.55, 95%CI: 1.01–2.38), poor self-reported health (OR: 3.31, 95%CI: 1.85–5.94), smoking (OR: 3.32, 95%CI: 1.06–10.33), ADL limitations (OR: 1.68, 95%CI: 1.09–2.61), and IADL limitations (OR: 2.39, 95%CI: 1.58–3.62) were significantly associated with increased depressive symptoms risk. Conversely, married and cohabiting (OR: 0.53, 95%CI: 0.29–0.97), marital satisfaction (OR: 0.29, 95%CI: 0.12–0.71), and life satisfaction (OR: 0.25, 95%CI: 0.14–0.46) show a decreased risk of depressive symptoms.

Among female patients, a nap time of ≥90 min (OR: 0.47, 95%CI: 0.23–0.96), married and cohabiting (OR: 0.39, 95%CI: 0.21–0.73), marital satisfaction (OR: 0.31, 95%CI: 0.16–0.61), and life satisfaction (OR: 0.20, 95%CI: 0.10–0.40) demonstrated a protective effect, while smoking, ADL limitations, and IADL limitations showed no significant association with depressive symptoms.

The study observed significant interactions between smoking and ADL limitations across different genders. Additionally, as shown in Table 3, all VIF values were below the commonly accepted threshold of 10, indicating the absence of significant multicollinearity in the logistic regression model.

### 3.5. Results of Random Forest

**Figure 2:**
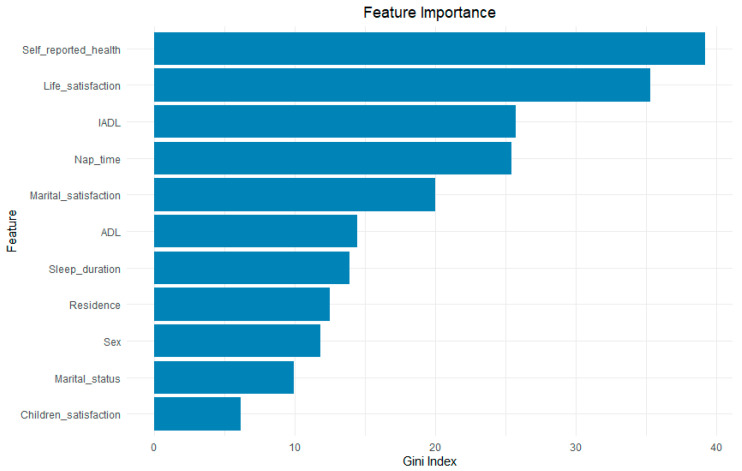
Ranking the importance of factors associated with depressive symptoms among CKD patients.

To further assess the importance of significant factors associated with depressive symptoms in middle-aged and older Chinese CKD patients, this study randomly allocated 70% of the total data as the training set and 30% as the test set. A random forest model was constructed, and the optimal hyperparameters (mtry = 3 and ntree = 500) were determined through grid search and 10-fold cross-validation. Feature importance was assessed based on the mean decrease in the Gini index, with higher values indicating a greater contribution of the variable to the classification performance. As shown in Supplementary Table S3, the AUC of the training set was 0.803, and the Accuracy, Sensitivity, Specificity, PPV, and NPV were 0.733, 0.796, 0.671, 0.702, and 0.772, respectively. For the test set, the AUC was 0.784, and the Accuracy, Sensitivity, Specificity, PPV, and NPV were 0.724, 0.814, 0.632, 0.692, and 0.770, respectively, indicating that the random forest model demonstrated good performance. As shown in Figure 2, self-reported health was the factor most strongly associated with depressive symptoms in this population. Other factors associated with depressive symptoms, ranked by the strength of their associations, included life satisfaction, IADL limitations, nap time, marital satisfaction, ADL limitations, sleep duration, residence, gender, marital status, and children satisfaction.

The performance metrics of the random forest model for the training and test sets among CKD patients of different genders are presented in Supplementary Tables S4 and S5. The results indicate that the random forest model demonstrated good performance. Among male CKD patients, the factors most strongly associated with the prevalence of depressive symptoms, in order of importance, were IADL, self-reported health, life satisfaction, ADL, residence, marital satisfaction, marital status, and smoking. Among female CKD patients, the two most significant factors mirrored those in the overall CKD population: self-reported health and life satisfaction. The subsequent factors included marital satisfaction, nap time, residence, and marital status. (As shown in Supplementary Figure S1, panel (A) presents the results for male patients, and panel (B)) presents the results for female patients).

## 4. Discussion

This study reveals that 50.35% of Chinese middle-aged and older CKD patients experience depressive symptoms, nearly twice the 24.10% prevalence in the general middle-aged and older population [1]. Existing research suggests that CKD is associated with the nervous system by disrupting neurotransmitter metabolism and balance, altering endocrine function, and influencing inflammatory factors, which are associated with depressive symptoms [43,44]. Additionally, this study found that the prevalence of depressive symptoms was 42.97% among men and 59.56% among women. Moreover, female patients with CKD had a significantly higher risk of developing depressive symptoms compared to their male counterparts. Epidemiological studies have shown that middle-aged and older women are more likely to experience depressive symptoms, potentially due to ovarian dysfunction and lower progesterone levels [45,46]. Additionally, women may exhibit greater emotional responsiveness to negative affect, such as sadness, which is associated with the observed gender differences in depressive symptoms [47]. These factors likely contribute to higher rates of depressive symptoms in women.

Additionally, within the dimension of demographic factors, self-reported health was also significantly associated with the occurrence of depressive symptoms. Self-reported health, a subjective measure recognized by the WHO, was strongly linked to depressive symptoms [48]. Studies conducted in Japan and France have shown that severe depressive symptoms are correlated with poor self-reported health [49]. CKD patients often experience comorbidities such as heart failure, encephalopathy, and infections, which are linked to disturbances in emotional regulation, suggesting that patients’ health conditions are a key factor associated with depressive symptoms [50,51]. These comorbidities may subject patients to sustained psychological stress, leading to concerns about their health, life dissatisfaction, and self-doubt, all of which contribute to negative emotions. Due to physiological decline and reduced self-care abilities in the elderly [52], their ability to perform ADLs and IADLs is often impaired. CKD patients often experience fatigue, which reduces their capacity to manage daily activities [53]. This decline is associated with various challenges and frustrations, reducing their sense of control over life, which correlates with feelings of helplessness and low self-worth. Over time, these negative emotions accumulate and are associated with poorer mental health in CKD patients. Previous research has found that limitations in ADLs and IADLs affect older adults’ mental health, with ADL limitations serving as a risk factor for depressive symptoms among older adults in China [39]. These findings align with the conclusions of this study.

Within the dimension of health behavior factors, nap time and sleep duration were significantly associated with depressive symptoms in CKD patients. Sleep is a crucial physiological process that regulates bodily functions and supports overall health [54]. Between 50.4% and 91.0% of CKD patients experience sleep problems. Insufficient sleep is associated with disruption of the body’s biological clock and various mental health issues [55,56]. Wang, X. et al. found that women who sleep less than 7 h per night have an increased risk of developing depressive symptoms [57]. Chronic sleep deprivation is associated with neurotransmitter imbalances in the brain and disruption of the secretion of mood-regulating chemicals. This, in turn, is associated with a reduced ability to manage negative emotions, accompanied by the accumulation of significant psychological distress [58]. Additionally, chronic sleep deprivation is associated with a weakened immune system and increased susceptibility to illness, factors also linked to depressive symptoms [59].

Among CKD patients in Stockholm, Sweden, the prevalence of depression among married individuals is 45.5%, compared to 11.2% among single individuals, which contrasts with the findings of this study [60]. However, a similar study in the US found that the risk of depressive symptoms among unmarried CKD patients is 1.26 times higher than that among married CKD patients, particularly among men, and that unmarried patients have a higher risk of mortality than their married peers [61]. Existing scholars attribute the impact of marital status on depressive symptoms to marital satisfaction [61]. This aligns with another finding of this study, which indicates that patients satisfied with their marriages had a 66.7% lower risk of developing depressive symptoms compared to those dissatisfied with their marriage. Higher marital satisfaction indicates strong marital relationships among CKD patients. Supportive partners are associated with mitigation of the effects of negative events, preservation of physical and emotional well-being, and a reduced likelihood of depressive symptoms [62]. Additionally, in terms of social network factors, this study found that CKD patients with higher life satisfaction have a lower risk of depressive symptoms. Among the elderly, life satisfaction is closely linked to psychological health [63]. Those with higher life satisfaction tend to have a more positive outlook, find joy in daily life, and feel greater control and accomplishment.

Additionally, in terms of living and working conditions, this study found that CKD patients in rural areas face a higher risk of depressive symptoms than those in urban areas, with female patients being especially vulnerable. Jin, W. et al. found that depressive symptoms are 1.37 times more prevalent among elderly individuals in rural areas than in urban areas, with rural women experiencing significantly higher levels of depressive symptoms than their urban counterparts [64]. The scarcity of medical resources, lower emphasis on mental health, and lack of effective mental health services in rural areas may contribute to higher rates of depressive symptoms compared to urban areas [65].

This study found that the impact of ADL limitations and smoking on depressive symptoms in CKD patients differs significantly by gender. Among male CKD patients, ADL limitations are associated with increased risk of depressive symptoms. These unhealthy habits are associated with an increased risk of chronic conditions, such as cardiovascular disease and liver cirrhosis, ultimately impairing ADL capabilities [66]. Additionally, due to traditional gender roles, men are overrepresented in high-risk occupations. Work-related injuries and accidents further limit ADLs in men. There is also a significant interaction between smoking and gender. In China, the proportion of male CKD patients who smoke is substantially higher than that of female patients [12]. Valdivielso et al. reported that among current smokers with CKD, as many as 75% were male [67]. The gender differences in smoking behavior may contribute to increased oxidative stress through activation of the sympathetic nervous system and the hypothalamic-pituitary–adrenal (HPA) axis. Notably, this oxidative stress response appears to be more pronounced in men, potentially due to endogenous testosterone levels, which may amplify the body’s stress response and exacerbate the severity of depressive symptoms [68,69]. In addition, smoking has traditionally been seen as a “male behavior”. Furthermore, since men more often work outdoors, they have more opportunities for social smoking.

The study also established an HEM to explain the multidimensional effects of depressive symptoms among middle-aged and older CKD patients in China. The findings of this study may serve as a theoretical basis for future health promotion efforts, supporting the development of comprehensive, multilevel interventions tailored to middle-aged and older adults with chronic kidney disease. Such interventions may help reduce the risk of depressive symptoms in this population in China.

## 5. Limitations

This study has several limitations. First, data on CKD, depressive symptoms, and key covariates in this study were based on participants’ self-reports. To enhance the reliability of these data, we applied strict inclusion and exclusion criteria, employed well-trained interviewers, and used validated instruments with established reliability and validity. Despite these efforts, the potential for recall bias and information misclassification cannot be fully ruled out. Future studies are encouraged to incorporate objective measures where possible to minimize such biases. Second, the participants in this study were middle-aged and older adults in China, which may limit the generalizability and applicability of the findings to other age groups, ethnicities, or healthcare settings. Sociocultural and healthcare characteristics specific to China may be associated with differences in the prevalence and correlates of depressive symptoms among individuals with CKD. As such, the applicability of these findings to other populations and regions should be considered with caution. Third, this study is limited by the inherent constraints of a cross-sectional design in inferring causality. Although we identified associations between depressive symptoms and certain factors among middle-aged and older Chinese adults with CKD, it remains undetermined whether depression leads to these factors or vice versa. Future longitudinal cohort studies are necessary to further explore these relationships and establish temporal sequences. Finally, depressive symptoms were assessed using the CES-D-10 scale, which captures symptoms experienced during the past week. A score of ≥10 on the CES-D-10 is considered clinically significant, indicating an elevated risk of depression and the need for further clinical evaluation. However, this method may introduce potential assessment bias.

## 6. Conclusions

Drawing on the HEM, this study examined factors associated with depressive symptoms among middle-aged and older CKD patients in China, using cross-sectional data from the 2018 CHARLS. Our findings indicate that female CKD patients had a higher prevalence of depressive symptoms than their male counterparts. Several factors were found to be associated with depressive symptoms in this population, including gender, self-reported health, nap time, sleep duration, marital status, marital satisfaction, children satisfaction, life satisfaction, place of residence, ADLs, and IADLs. Notably, the associations of smoking and ADLs with depressive symptoms differed by gender. Among all factors, self-reported health and life satisfaction were most strongly associated with depressive symptoms. These findings underscore associations relevant to the mental health needs of middle-aged and older Chinese CKD patients and provide valuable insights for targeted interventions and policy recommendations.

## Figures and Tables

**Table 1:** Sociodemographic characteristics of middle-aged and older patients with CKD in China, n (%).

Variables	Total (n = 1422)	Male (n = 789)	Female (n = 633)
**Demographic factors**			
Age, n (%)			
45–60	335 (23.56)	166 (21.04)	169 (26.70)
>60	1087 (76.44)	623 (78.96)	464 (73.30)
Ethnicity, n (%)			
Non-Han	118 (8.30)	59 (7.48)	59 (9.32)
Han	1304 (91.70)	730 (92.52)	574 (90.68)
Self-reported Health, n (%)			
Good	147 (10.34)	104 (13.18)	43 (6.79)
Fair	645 (45.36)	364 (46.13)	281 (44.39)
Poor	630 (44.30)	321 (40.68)	309 (48.82)
Hypertension, n (%)			
No	1250 (87.90)	681 (86.31)	569 (89.89)
Yes	172 (12.10)	108 (13.69)	64 (10.11)
Diabetes, n (%)			
No	1310 (92.12)	735 (93.16)	575 (90.84)
Yes	112 (7.88)	54 (6.84)	58 (9.16)
Dyslipidemia, n (%)			
No	894 (62.87)	501 (63.50)	393 (62.09)
Yes	528 (37.13)	288 (36.50)	240 (37.91)
Stroke, n (%)			
No	1266 (89.03)	694 (87.96)	572 (90.36)
Yes	156 (10.97)	95 (12.04)	61 (9.64)
Depressive Symptoms, n (%)			
No	706(49.65)	450(57.03)	256(40.44)
Yes	716(50.35)	339(42.97)	377(59.56)
ADLs, n (%)			
No	1048 (73.70)	613 (77.69)	435 (68.72)
Yes	374 (26.30)	176 (22.31)	198 (31.28)
IADLs, n (%)			
No	963 (67.72)	583 (73.89)	380 (60.03)
Yes	459 (32.28)	206 (26.11)	253 (39.97)
**Health behavior factors**			
Smoking, n (%)			
No	1400 (98.45)	771 (97.72)	629 (99.37)
Yes	22 (1.55)	18 (2.28)	4 (0.63)
Drinking, n (%)			
No	932 (65.54)	380 (48.16)	552 (87.20)
Yes	490 (34.46)	409 (51.84)	81 (12.80)
Nap Time, n (%)			
0	584 (41.07)	286 (36.25)	298 (47.08)
<30	277 (19.48)	153 (19.39)	124 (19.59)
30–89	353 (24.82)	215 (27.25)	138 (21.80)
≥90	208 (14.63)	135 (17.11)	73 (11.53)
Sleep Duration, n (%)			
<7	1096 (77.07)	571 (72.37)	525 (82.94)
7–9	278 (19.55)	183 (23.19)	95 (15.01)
>9	48 (3.38)	35 (4.44)	13 (2.05)
Activity, n (%)			
Inactive	635 (44.66)	352 (44.61)	283 (44.71)
Active	787 (55.34)	437 (55.39)	350 (55.29)
**Social network factors**			
Marital Status, n (%)			
Other	153 (10.76)	62 (7.86)	91 (14.38)
Married and Cohabiting	1269 (89.24)	727 (92.14)	542 (85.62)
Marital Satisfaction, n (%)			
Dissatisfied	164 (11.53)	46 (5.83)	118 (18.64)
Satisfied	1258 (88.47)	743 (94.17)	515 (81.36)
Children Satisfaction, n (%)			
Dissatisfied	81 (5.70)	47 (5.96)	34 (5.37)
Satisfied	1341 (94.30)	742 (94.04)	599 (94.63)
Life Satisfaction, n (%)			
Dissatisfied	228 (16.03)	100 (12.67)	128 (20.22)
Satisfied	1194 (83.97)	689 (87.33)	505 (79.78)
**Living and working conditions factors**			
Place of Residence, n (%)			
Urban	421 (29.61)	239 (30.29)	182 (28.75)
Rural	1001 (70.39)	550 (69.71)	451 (71.25)
Type of Residence, n (%)			
Private Residence	1382 (97.19)	765 (96.96)	617 (97.47)
Other	40 (2.81)	24 (3.04)	16 (2.53)
Education Level, n (%)			
Illiterate	226 (15.89)	64 (8.11)	162 (25.59)
Primary School or Below	663 (46.62)	370 (46.89)	293 (46.29)
Above Primary School	533 (37.48)	355 (44.99)	178 (28.12)
**Social policy factors**			
Insurance, n (%)			
No	26 (1.83)	11 (1.39)	15 (2.37)
Yes	1396 (98.17)	778 (98.61)	618 (97.63)
Pension, n (%)			
No	1099 (77.29)	575 (72.88)	524 (82.78)
Yes	323 (22.71)	214 (27.12)	109 (17.22)

Note: ADLs: activities of daily living, IADLs: instrumental activities of daily living.

**Table 2:** Multivariate logistic regression analysis of depressive symptoms in Chinese middle-aged and older CKD patients.

Variables	OR (95%CI)	*p*	VIF
**Demographic factors**			
Gender			1.405
Male	1.00 (Reference)		
Female	1.40 (1.05–1.87)	0.021	
Age			1.122
45–60	1.00 (Reference)		
≥60	0.97 (0.72–1.31)	0.832	
Ethnicity			1.024
Non-Han	1.00 (Reference)		
Han	1.18 (0.76–1.84)	0.466	
Self-reported Health			1.339
Good	1.00 (Reference)		
Fair	1.35 (0.87–2.09)	0.178	
Poor	3.04 (1.92–4.81)	<0.001	
Hypertension			1.044
No	1.00 (Reference)		
Yes	0.99 (0.68–1.44)	0.946	
Diabetes			1.041
No	1.00 (Reference)		
Yes	0.66 (0.41–1.06)	0.083	
Dyslipidemia			1.141
No	1.00 (Reference)		
Yes	1.10 (0.84–1.44)	0.488	
Stroke			1.078
No	1.00 (Reference)		
Yes	1.47 (0.97–2.23)	0.066	
ADLs			1.396
No	1.00 (Reference)		
Yes	1.48 (1.08–2.05)	0.016	
IADLs			1.450
No	1.00 (Reference)		
Yes	1.98 (1.46–2.68)	<0.001	
**Health behavior factors**			
Smoking			1.036
No	1.00 (Reference)		
Yes	2.13 (0.76–5.99)	0.153	
Drinking			1.284
No	1.00 (Reference)		
Yes	1.00 (0.75–1.34)	1.000	
Nap Time			1.121
<30	1.00 (Reference)		
0	0.97 (0.69–1.36)	0.845	
30–89	0.81 (0.56–1.17)	0.264	
≥90	0.57 (0.37–0.87)	0.010	
Sleep Duration			1.083
7–9	1.00 (Reference)		
<7	1.37 (1.01–1.88)	0.048	
>9	1.11 (0.54–2.25)	0.782	
Activity			1.092
Inactive	1.00 (Reference)		
Active	1.03 (0.80–1.33)	0.803	
**Social network factors**			
Marital Satisfaction			1.274
Dissatisfied	1.00 (Reference)		
Satisfied	0.33 (0.20–0.56)	<0.001	
Children Satisfaction			1.164
Dissatisfied	1.00 (Reference)		
Satisfied	0.39 (0.19–0.77)	0.007	
Life Satisfaction			1.253
Dissatisfied	1.00 (Reference)		
Satisfied	0.22 (0.14–0.35)	<0.001	
Marital Status			1.056
Other	1.00 (Reference)		
Married and Cohabiting	0.46 (0.30–0.70)	<0.001	
**Living and working conditions factors**			
Place of Residence			1.459
Urban	1.00 (Reference)		
Rural	1.83 (1.32–2.54)	<0.001	
Type of Residence			1.047
Private Residence	1.00 (Reference)		
Other	0.73 (0.33–1.58)	0.420	
Education Level			1.379
Illiterate	1.00 (Reference)		
Primary School or Below	1.01 (0.69–1.47)	0.951	
Above Primary School	0.89 (0.59–1.35)	0.578	
**Social policy factors**			
Insurance			1.021
No	1.00 (Reference)		
Yes	0.77 (0.29–2.03)	0.590	
Pension			1.555
No	1.00 (Reference)		
Yes	0.87 (0.61–1.25)	0.458	

Note: OR: odds ratio, CI: confidence interval, ADLs: activities of daily living, IADLs: instrumental activities of daily living, VIF: variance inflation factor.

**Table 3:** Logistic regression analysis of gender differences in depressive symptoms and associated factors among CKD patients.

Variables	Male			Female			Coefficient (B)
	*p*	OR (95%CI)	VIF	*p*	OR (95%CI)	VIF	
**Demographic factors**							
Age			1.123			1.182	
45–60		1.00 (Reference)			1.00 (Reference)		
>60	0.457	1.17 (0.77–1.79)		0.230	0.75 (0.47–1.20)		−0.400
Ethnicity			1.043			1.046	
Non-Han		1.00 (Reference)			1.00 (Reference)		
Han	0.844	0.94 (0.51–1.75)		0.274	1.44 (0.75–2.75)		0.450
Self-reported Health			1.350			1.382	
Good		1.00 (Reference)			1.00 (Reference)		
Fair	0.135	1.53 (0.88–2.67)		0.636	1.20 (0.57–2.54)		−0.102
Poor	<0.001	3.31 (1.85–5.94)		0.007	2.97 (1.35–6.53)		−0.042
Hypertension			1.057			1.079	
No		1.00 (Reference)			1.00 (Reference)		
Yes	0.531	0.86 (0.53–1.39)		0.333	1.38 (0.72–2.65)		0.535
Diabetes			1.039			1.088	
No		1.00 (Reference)			1.00 (Reference)		
Yes	0.101	0.56 (0.28–1.12)		0.300	0.70 (0.35–1.38)		0.419
Dyslipidemia			1.210			1.119	
No		1.00 (Reference)			1.00 (Reference)		
Yes	0.406	0.85 (0.59–1.24)		0.061	1.49 (0.98–2.27)		0.475
Stroke			1.094			1.110	
No		1.00 (Reference)			1.00 (Reference)		
Yes	0.343	1.28 (0.77–2.16)		0.070	2.02 (0.94–4.34)		0.400
ADLs			1.364			1.428	
No		1.00 (Reference)			1.00 (Reference)		
Yes	0.020	1.68 (1.09–2.61)		0.359	1.26 (0.77–2.05)		−0.403 *
IADLs			1.395			1.506	
No		1.00 (Reference)			1.00 (Reference)		
Yes	<0.001	2.39 (1.58–3.62)		0.051	1.58 (1.00–2.50)		−0.454
**Health behavior factors**							
Smoking			1.046			1.034	
No		1.00 (Reference)			1.00 (Reference)		
Yes	0.039	3.32 (1.06–10.33)		0.543	0.40 (0.02–7.69)		−2.066 *
Drinking			1.091			1.074	
No		1.00 (Reference)			1.00 (Reference)		
Yes	0.878	1.03 (0.73–1.44)		0.990	1.00 (0.55–1.85)		−0.064
Nap Time			1.137			1.179	
<30		1.00 (Reference)			1.00 (Reference)		
0	0.689	0.91 (0.57–1.45)		0.958	0.99 (0.58–1.67)		−0.071
30–89	0.309	0.78 (0.48–1.27)		0.488	0.81 (0.44–1.49)		−0.053
≥90	0.076	0.61 (0.35–1.05)		0.039	0.47 (0.23–0.96)		−0.390
Sleep Duration			1.099			1.154	
7–9		1.00 (Reference)			1.00 (Reference)		
<7	0.296	1.24 (0.83–1.86)		0.144	1.49 (0.87–2.55)		−0.154
>9	0.361	1.50 (0.63–3.57)		0.415	0.57 (0.15–2.21)		−1.328
Activity			1.125			1.079	
Inactive		1.00 (Reference)			1.00 (Reference)		
Active	0.831	0.96 (0.69–1.35)		0.370	1.20 (0.81–1.79)		0.211
**Social network factors**							
Marital Status			1.076		1.00 (Reference)	1.055	
Other		1.00 (Reference)			1.00 (Reference)		
Married and Cohabiting	0.039	0.53 (0.29–0.97)		0.003	0.39 (0.21–0.73)		−0.211
Marital Satisfaction			1.172			1.335	
Dissatisfied		1.00 (Reference)			1.00 (Reference)		
Satisfied	0.007	0.29 (0.12–0.71)		<0.001	0.31 (0.16–0.61)		0.149
Children Satisfaction			1.143			1.243	
Dissatisfied		1.00 (Reference)			1.00 (Reference)		
Satisfied	0.066	0.48 (0.22–1.05)		0.092	0.24 (0.05–1.26)		−0.575
Life Satisfaction			1.268			1.274	
Dissatisfied		1.00 (Reference)			1.00 (Reference)		
Satisfied	<0.001	0.25 (0.14–0.46)		<0.001	0.20 (0.10–0.40)		−0.272
**Living and working conditions factors**							
Place of Residence			1.438			1.596	
Urban		1.00 (Reference)			1.00 (Reference)		
Rural	0.047	1.55 (1.01–2.38)		0.002	2.34 (1.38–3.97)		0.217
Type of Residence			1.080			1.067	
Private Residence		1.00 (Reference)			1.00 (Reference)		
Other	0.757	1.17 (0.43–3.20)		0.130	0.37 (0.10–1.34)		−1.173
Education Level			1.282			1.431	
Illiterate		1.00 (Reference)			1.00 (Reference)		
Primary School or Below	0.533	1.22 (0.65–2.28)		0.523	0.85 (0.51–1.41)		−0.126
Above Primary School	0.454	1.28 (0.67–2.46)		0.088	0.59 (0.33–1.08)		−0.394
**Social policy factors**							
Insurance			1.024			1.034	
No		1.00 (Reference)			1.00 (Reference)		
Yes	0.375	0.54 (0.14–2.10)		0.907	0.92 (0.24–3.55)		0.626
Pension			1.509			1.654	
No		1.00 (Reference)			1.00 (Reference)		
Yes	0.201	0.74 (0.47–1.17)		0.676	1.14 (0.61–2.15)		0.202

Note: *: *p* < 0.05, OR: odds ratio, CI: confidence interval, ADLs: activities of daily living, IADLs: instrumental activities of Daily living, VIF: variance inflation factor.

## Data Availability

The CHARLS data are available at http://charls.pku.edu.cn/ (accessed on 8 August 2022).

## Supplementary Materials

The following supporting information can be downloaded at: https://www.mdpi.com/article/10.3390/healthcare13161951/s1, Table S1. Questionnaire items and classification criteria of covariates. Table S2. Description of the basic demographic characteristics of CKD patients by gender and depressive symptoms status. Table S3. Random forest model performance metrics among Chinese middle-aged and older adults with CKD. Table S4. Random forest model performance metrics among Chinese middle-aged and older male CKD patients. Table S5. Random forest model performance metrics among Chinese middle-aged and older female CKD patients. Figure S1. Ranking the importance of factors associated with depressive symptoms among middle-aged and older Chinese male and female CKD patients. Note: (A) males; (B) females.
