# Supplementary material for: Depressive Symptoms and Associated Factors Among Middle-Aged and Older Patients with Chronic Kidney Disease: Gender Differences Based on a Health Ecological Model
Source: Healthcare (Basel). 2025 Aug 9;13(16):1951. doi: 10.3390/healthcare13161951 (PMC12385398; doi:10.3390/healthcare13161951)
Supplement: Supplementary file 1 [file healthcare-13-01951-s001.zip › healthcare-3744575-supplementary.pdf]

Supplementary Materials

Table S1. Questionnaire items and classification criteria of covariates.

**Demographic factors**		
Age	What's your date of birth on ID card or Household register?	0= 45 - 60<br>1= > 60
Gender	Interviewer records the Respondent's gender.	0 = Male<br>1 = Female
Ethnicity	Are you Han or Ethnic Minorities?	0 = Non-Han<br>1 = Han
Self-reported Health	How satisfied are you with your health?	0 = Good<br>1 = Fair<br>2 = Poor
Hypertension	Have you been diagnosed with Hypertension by a doctor?	0 = No<br>1 = Yes
Diabetes	Have you been diagnosed with Diabetes or high blood sugar by a doctor?	0 = No<br>1 = Yes
Dyslipidemia	Have you been diagnosed with Dyslipidemia (elevation of low density lipoprotein, triglycerides (TGs),and total cholesterol, or a low high density lipoprotein level) by a doctor?	0 = No<br>1 = Yes
Stroke	Have you been diagnosed with Stroke by a doctor?	0 = No<br>1 = Yes
Depressive Symptoms	/	0 = No<br>1 = Yes
Activities of Daily Living	/	0 = No<br>1 = Yes
Instrumental Activities of Daily Living	/	0 = No<br>1 = Yes
**Health behavior factors**		
Smoking	Have you ever chewed tobacco, smoked a pipe, smoked self-rolled cigarettes, or smoked cigarettes/cigars?	0 = No<br>1 = Yes
Drinking	Did you drink any alcoholic beverages, such as beer, wine, or liquor in the past year? How often?	0 = No<br>1 = Yes
Sleep Duration	During the past month, how many hours of actual sleep did you get at night (average hours for one night)? (This may be shorter than the number of hours you spend in bed.)	0 = < 7<br>1 = 7-9<br>2 = ＞9
Nap Time	During the past month, how long did you take a nap after lunch?	0 = 0<br>1 =<30<br>2 = 30 - 89

		3= ≥90
Activity	Have you done any of these activities in the last month? (Check all that apply)	0 = Inactive<br>1 = Active
**Social network factors**		
Marital Satisfaction	How satisfied are you with your marriage (relationship with spouse)?	0 = Dissatisfied<br>1 = Satisfied
Marital Status	What is your marital status?	0 = Other<br>1 = Married and Cohabiting
Children Satisfaction	How satisfied are you with your relationship with children?	0 = Dissatisfied<br>1 = Satisfied
Life Satisfaction	How satisfied are you with your life-as-a-whole?	0 = Dissatisfied<br>1 = Satisfied
**Living and working conditions factors**		
Education Level	What's the highest level of education your have now (not including adult education)?	0 = Illiterate<br>1 = Primary School or Below<br>2 = Above Primary School
Place of Residence	Was your address BB001_W3 in the village or city/town?	0 = Urban<br>1 = Rural
Type of Residence	What was the type of your address, BB000_W3 in [ZIWTime]?	0 = Private Residence<br>1 = Other
**Social policy factors**		
Pension	Do you currently receive, expect to receive, or contribute to the pension for public servants, or pension for public institution employees, or basic pension for enterprise employees?	0 = No<br>1 = Yes
Insurance	Are you the policy holder/primary beneficiary of any of the types of health insurance listed below? (circle all that apply)	0 = No<br>1 = Yes

Table S2. Description of the basic demographic characteristics of CKD patients by gender and depressive symptoms status.

Variables	Male				Female			
	Total	No depressive symptoms	Depressive symptoms	Statistic/P	Total	No depressive symptoms	Depressive symptoms	Statistic/P
n (%)	789 (100%)	450 (57.03%)	339 (42.97%)		633 (100%)	256 (40.44%)	377 (59.56%)	
**Demographic factors**								
Age, n (%)				3.32 (0.069)				0.18 (0.667)
45-60	166 (21.04)	105 (23.33)	61 (17.99)		169 (26.70)	66 (25.78)	103 (27.32)	
> 60	623 (78.96)	345 (76.67)	278 (82.01)		464 (73.30)	190 (74.22)	274 (72.68)	
Ethnicity, n (%)				0.03 (0.850)				1.33 (0.249)
Non-Han	59 (7.48)	33 (7.33)	26 (7.67)		59 (9.32)	28 (10.94)	31 (8.22)	
Han	730 (92.52)	417 (92.67)	313 (92.33)		574 (90.68)	228 (89.06)	346 (91.78)	
Self-reported Health, n (%)				70.03 (<.001)				64.82 (<.001)
Good	104 (13.18)	79 (17.56)	25 (7.37)		43 (6.79)	28 (10.94)	15 (3.98)	
Fair	364 (46.13)	244 (54.22)	120 (35.40)		281 (44.39)	152 (59.38)	129 (34.22)	
Poor	321 (40.68)	127 (28.22)	194 (57.23)		309 (48.82)	76 (29.69)	233 (61.80)	
Hypertension, n (%)				0.50 (0.476)				0.26 (0.613)
No	681 (86.31)	385 (85.56)	296 (87.32)		569 (89.89)	232 (90.62)	337 (89.39)	
Yes	108 (13.69)	65 (14.44)	43 (12.68)		64 (10.11)	24 (9.38)	40 (10.61)	
Diabetes, n (%)				0.83 (0.362)				0.51 (0.475)
No	735 (93.16)	416 (92.44)	319 (94.10)		575 (90.84)	230 (89.84)	345 (91.51)	
Yes	54 (6.84)	34 (7.56)	20 (5.90)		58 (9.16)	26 (10.16)	32 (8.49)	
Dyslipidemia, n (%)				0.20 (0.626)				7.19 (0.007)
No	501 (63.50)	289 (64.22)	212 (62.54)		393 (62.09)	175 (68.36)	218 (57.82)	

Yes	288 (36.50)	161 (35.78)	127 (37.46)		240 (37.91)	81 (31.64)	159 (42.18)	
Stroke, n (%)				5.06 (0.024)				12.09 (<.001)
No	694 (87.96)	406 (90.22)	288 (84.96)		572 (90.36)	244 (95.31)	328 (87.00)	
Yes	95 (12.04)	44 (9.78)	51 (15.04)		61 (9.64)	12 (4.69)	49 (13.00)	
ADLs, n (%)				51.10 (<.001)				27.60 (<.001)
No	613 (77.69)	391 (86.89)	222 (65.49)		435 (68.72)	206 (80.47)	229 (60.74)	
Yes	176 (22.31)	59 (13.11)	117 (34.51)		198 (31.28)	50 (19.53)	148 (39.26)	
IADLs, n (%)				68.34 (<.001)				38.07 (<.001)
No	583 (73.89)	383 (85.11)	200 (59.00)		380 (60.03)	191 (74.61)	189 (50.13)	
Yes	206 (26.11)	67 (14.89)	139 (41.00)		253 (39.97)	65 (25.39)	188 (49.87)	
**Health behavior factors**								
Nap Time, n (%)				5.69 (0.128)				7.86 (0.049)
0	286 (36.25)	149 (33.11)	137 (40.41)		298 (47.08)	111 (43.36)	187 (49.60)	
<30	153 (19.39)	86 (19.11)	67 (19.76)		124 (19.59)	52 (20.31)	72 (19.10)	
30-89	215 (27.25)	133 (29.56)	82 (24.19)		138 (21.80)	53 (20.70)	85 (22.55)	
≥90	135 (17.11)	82 (18.22)	53 (15.63)		73 (11.53)	40 (15.62)	33 (8.75)	
Sleep Duration, n (%)				5.25 (0.073)				16.42 (<.001)
< 7	571 (72.37)	316 (70.22)	255 (75.22)		525 (82.94)	194 (75.78)	331 (87.80)	
7-9	183 (23.19)	117 (26.00)	66 (19.47)		95 (15.01)	53 (20.70)	42 (11.14)	
＞9	35 (4.44)	17 (3.78)	18 (5.31)		13 (2.05)	9 (3.52)	4 (1.06)	
Smoking, n (%)				2.47 (0.116)				0.00 (1.000)
No	771 (97.72)	443 (98.44)	328 (96.76)		629 (99.37)	254 (99.22)	375 (99.47)	
Yes	18 (2.28)	7 (1.56)	11 (3.24)		4 (0.63)	2 (0.78)	2 (0.53)	
Drinking, n (%)				4.49				0.62

				(0.034)				(0.432)
No	380 (48.16)	202 (44.89)	178 (52.51)		552 (87.20)	220 (85.94)	332 (88.06)	
Yes	409 (51.84)	248 (55.11)	161 (47.49)		81 (12.80)	36 (14.06)	45 (11.94)	
Activity, n (%)				5.88 (0.015)				0.53 (0.468)
Inactive	352 (44.61)	184 (40.89)	168 (49.56)		283 (44.71)	110 (42.97)	173 (45.89)	
Active	437 (55.39)	266 (59.11)	171 (50.44)		350 (55.29)	146 (57.03)	204 (54.11)	
**Social network factors**								
Marital Status, n (%)				3.87 (0.049)				10.15 (0.001)
Other	62 (7.86)	28 (6.22)	34 (10.03)		91 (14.38)	23 (8.98)	68 (18.04)	
Married and Cohabiting	727 (92.14)	422 (93.78)	305 (89.97)		542 (85.62)	233 (91.02)	309 (81.96)	
Marital Satisfaction, n (%)				31.33 (<.001)				43.52 (<.001)
Dissatisfied	46 (5.83)	8 (1.78)	38 (11.21)		118 (18.64)	16 (6.25)	102 (27.06)	
Satisfied	743 (94.17)	442 (98.22)	301 (88.79)		515 (81.36)	240 (93.75)	275 (72.94)	
Children Satisfaction, n (%)				17.60 (<.001)				17.82 (<.001)
Dissatisfied	47 (5.96)	13 (2.89)	34 (10.03)		34 (5.37)	2 (0.78)	32 (8.49)	
Satisfied	742 (94.04)	437 (97.11)	305 (89.97)		599 (94.63)	254 (99.22)	345 (91.51)	
Life Satisfaction, n (%)				67.60 (<.001)				67.57 (<.001)
Dissatisfied	100 (12.67)	19 (4.22)	81 (23.89)		128 (20.22)	11 (4.30)	117 (31.03)	
Satisfied	689 (87.33)	431 (95.78)	258 (76.11)		505 (79.78)	245 (95.70)	260 (68.97)	
**Living and working conditions factors**								
Place of Residence, n (%)				11.52 (<.001)				24.03 (<.001)
Urban	239 (30.29)	158 (35.11)	81 (23.89)		182 (28.75)	101 (39.45)	81 (21.49)	
Rural	550 (69.71)	292 (64.89)	258 (76.11)		451 (71.25)	155 (60.55)	296 (78.51)	
Type of Residence, n (%)				0.50 (0.480)				1.70 (0.192)
Private Residence	765 (96.96)	438 (97.33)	327 (96.46)		617	247	370	

					(97.47)	(96.48)	(98.14)	
Other	24 (3.04)	12 (2.67)	12 (3.54)		16 (2.53)	9 (3.52)	7 (1.86)	
Education Level, n (%)				5.79 (0.055)				18.31 (<.001)
Illiterate	64 (8.11)	33 (7.33)	31 (9.14)		162 (25.59)	52 (20.31)	110 (29.18)	
Primary School or Below	370 (46.89)	198 (44.00)	172 (50.74)		293 (46.29)	109 (42.58)	184 (48.81)	
Above Primary School	355 (44.99)	219 (48.67)	136 (40.12)		178 (28.12)	95 (37.11)	83 (22.02)	
**Social policy factors**								
Insurance, n (%)				0.23 (0.635)				0.32 (0.570)
No	11 (1.39)	5 (1.11)	6 (1.77)		15 (2.37)	5 (1.95)	10 (2.65)	
Yes	778 (98.61)	445 (98.89)	333 (98.23)		618 (97.63)	251 (98.05)	367 (97.35)	
Pension, n (%)				16.28 (<.001)				11.66 (<.001)
No	575 (72.88)	303 (67.33)	272 (80.24)		524 (82.78)	196 (76.56)	328 (87.00)	
Yes	214 (27.12)	147 (32.67)	67 (19.76)		109 (17.22)	60 (23.44)	49 (13.00)	

Note: χ^2: chi-square test, ADLs: activities of daily living, IADLs: instrumental activities of daily living.

Table S3. Random forest model performance metrics among Chinese middle-aged and older adults with CKD.

Data	AUC (95%CI)	Accuracy (95%CI)	Sensitivity (95%CI)	Specificity (95%CI)	PPV (95%CI)	NPV (95%CI)	Cut off
Train	0.803 (0.776-0.830)	0.733 (0.704-0.760)	0.796 (0.761 - 0.832)	0.671 (0.630 - 0.712)	0.702 (0.664 - 0.740)	0.772 (0.732 - 0.811)	0.524
Test	0.784 (0.741-0.827)	0.724 (0.679-0.766)	0.814 (0.762 - 0.866)	0.632 (0.567 - 0.697)	0.692 (0.635 - 0.749)	0.770 (0.708 - 0.833)	0.524

Note: PPV: positive predictive value, NPV: negative predictive value.

Table S4. Random forest model performance metrics among Chinese middle-aged and older male CKD patients.

Data	AUC (95%CI)	Accuracy (95%CI)	Sensitivity (95%CI)	Specificity (95%CI)	PPV (95%CI)	NPV (95%CI)	Cut off
Train	0.754 (0.713-0.795)	0.701 (0.661-0.739)	0.707 (0.658 - 0.756)	0.692 (0.631 - 0.753)	0.775 (0.728 - 0.822)	0.612 (0.552 - 0.672)	0.397
Test	0.827 (0.775-0.879)	0.759 (0.700-0.812)	0.782 (0.707 - 0.856)	0.737 (0.658 - 0.817)	0.750 (0.674 - 0.826)	0.770 (0.692 - 0.848)	0.397

Note: PPV: positive predictive value, NPV: negative predictive value.

Table S5. Random forest model performance metrics among Chinese middle-aged and older female CKD patients.

Data	AUC (95%CI)	Accuracy (95%CI)	Sensitivity (95%CI)	Specificity (95%CI)	PPV (95%CI)	NPV (95%CI)	Cut off
Train	0.809 (0.769-0.849)	0.734 (0.690-0.774)	0.824 (0.770 - 0.879)	0.667 (0.609 - 0.725)	0.646 (0.585 - 0.706)	0.837 (0.787 - 0.888)	0.626
Test	0.845 (0.790-0.899)	0.732 (0.663-0.793)	0.809 (0.715 - 0.902)	0.689 (0.606 - 0.771)	0.591 (0.491 - 0.691)	0.866 (0.798 - 0.934)	0.626

Note: PPV: positive predictive value, NPV: negative predictive value.

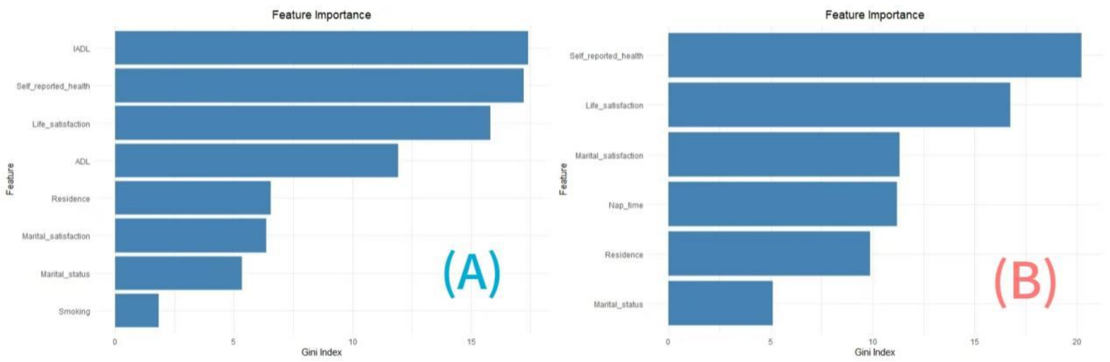

Figure S1. Ranking the importance of factors associated with depressive symptoms among middle-aged and older Chinese male and female CKD patients.

Note: (A) males; (B) females.
